# The Role of Olfaction in Dogs: Evolution, Biology, and Human-Oriented Work

**DOI:** 10.3390/ani16030427

**Published:** 2026-01-29

**Authors:** Iwona Kowalczyk-Jabłońska, Paulina Jundziłł-Bogusiewicz, Tadeusz Kaleta

**Affiliations:** Department of Animal Genetics and Conservation, Institute of Animal Sciences, Warsaw University of Life Sciences-SGGW, 8 Ciszewskiego Str., 02-786 Warsaw, Poland

**Keywords:** canine olfaction, olfactory anatomy, scent detection dogs, working dogs, olfactory neurobiology, selective breeding, domestication effects, behavioral predispositions, detection dog performance, microbiome and olfaction

## Abstract

Dogs show exceptional olfactory sensitivity and are widely used in medical, rescue, military, and forensic applications, yet the determinants of individual and breed-level scent-work performance remain incompletely characterized. This review integrates evidence from the anatomy and physiology of the canine olfactory organ, neurobiological mechanisms of odor transduction and coding, and links between olfaction, memory, and emotion, alongside molecular genetics, evolution, domestication, and selective breeding. We synthesize findings indicating that complex nasal turbinates and specialized airflow patterns enhance odorant capture, while olfactory bulb circuitry and downstream connections to limbic and frontal networks support discrimination, learning, and affective modulation. Comparative and breed-focused studies suggest that skull morphology and breeding priorities can alter olfactory capacity, with shortened nasal anatomy associated with reduced functional potential in some lines. In applied contexts, detection success is strongly shaped by behavioral traits such as motivation, persistence, independence, and reward value, as well as by physical condition and environmental stressors that can impair search efficiency. Emerging literature further suggests that the gastrointestinal and upper airway microbiome, together with diet, housing, temperature, and workload, may influence sensory and cognitive readiness, although direct causal links to detection outcomes remain limited. Overall, canine olfactory performance reflects interactions among genetic–anatomical capacity, neurobehavioral factors, and environment, underscoring the value of standardized selection, training, welfare management, and future integrative research.

## Simple Summary

A dog’s sense of smell is one of its most important biological abilities and underpins many tasks performed with humans, including finding missing persons, detecting hazardous materials, and supporting medical screening. Despite major progress, it is still not fully clear why some dogs perform scent work better than others and how body structure, physical fitness, stress, fatigue, and living conditions influence this ability. This review brings together current knowledge on how a dog’s nose and brain support smell detection, how genes contribute to odor sensitivity, how domestication and selective breeding have shaped breed differences, and how training uses natural hunting-related motivation to produce reliable scent work. The reviewed evidence indicates that performance depends on both “hardware” (nose and brain structure) and “software” (motivation, learning, and resistance to distraction), and that welfare and health are essential for maintaining high sensory performance. Better understanding of these factors can improve the selection and training of working and assistance dogs, reduce avoidable performance losses, and support safe, ethical, and effective deployments that benefit public safety, medicine, and society.

## 1. Introduction

The sense of smell is a fundamental part of dog biology and behavior and plays a crucial role in cooperation between dogs and humans. Dogs have a much more sensitive sense of smell than people, which makes them valuable in detecting dangerous materials, biological substances, missing persons, certain diseases, and in rescue operations. As biomedical and brain sciences develop, there is a growing need to better understand how dogs detect smells so effectively and which factors can influence this ability [1]. Particular attention is now given to the community of microorganisms living in the dog’s body and its influence on the nervous and immune systems, as well as to the relationship between a dog’s physical condition and the effectiveness of smell-based work in real operational conditions. Current scientific studies show that knowledge about the structure of the nose and brain related to smell has expanded considerably, but the chemical, genetic, and environmental processes that may determine a dog’s natural suitability for smell detection are still not fully understood. This is especially important for working dogs, in which physical strain, environmental stress, fatigue, and changes in body microorganisms may reduce sensory performance. The aim of this review is to integrate knowledge from brain science, genetics, evolution, behavior, and practical dog training, and to present different aspects of the practical use of dogs’ sense of smell in medical, rescue, military, and forensic activities.

## 2. Anatomy and Physiology of the Olfactory Organ

The sense of smell in dogs is one of the most highly developed among all mammals, which is reflected in the anatomical structure of the nose and in the organization of the central nervous system. The canine nasal structure is highly complex and adapted to an exceptional olfactory capability. It consists of an external part (the nasal planum) and a complex internal cavity lined with a richly vascularized and highly folded mucous membrane containing numerous olfactory receptors.

In Canidae, the olfactory organ is divided into two main components: the main olfactory system (MOS—main olfactory system), which detects volatile odorant substances and is responsible for the conscious perception of odors, and Jacobson’s organ, the vomeronasal organ (VNO—vomeronasal organ), which is responsible for the detection of social and reproductive chemical compounds that are not consciously perceived. The VNO functions in parallel, cooperating with hypothalamic and limbic system centers to modulate social behaviors [2].

**Figure 1:**
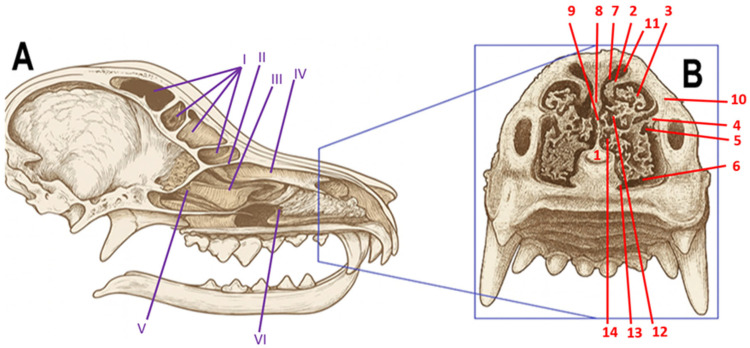
The canine olfactory organ longitudinal section (**A**) (I—Frontal sinus, II—Ethmoid bone, III—Endoturbinates, IV—Plica recta, V—Posterior nostrils, VI—Ventral turbinates) and transverse section (**B**) (1—Vomer, 2—Endoturbinate I (dorsal nasal concha), 3—Endoturbinate II, 4—Concha crest, 5—Ectoturbinate, 6—Ventral nasal meatus, 7—Ectoturbinate, 8—Ethmoid bone, 9—Bony nasal septum, 10—Maxilla, 11—Dorsal nasal meatus, 12—Common nasal meatus, 13,14—Endoturbinates) (author’s own illustration).

Dogs possess a markedly more sensitive sense of smell than humans, having between 125 and 300 million olfactory receptors, whereas humans have only about 5 million [3,4,5,6,7]. In addition, dogs have a highly developed turbinate system, including both nasal and ethmoidal turbinates. Within the nasal cavity, three main turbinates can be distinguished: dorsal, middle, and ventral, as well as numerous ethmoidal bony projections—endoturbinates and ectoturbinates—which form a complex spatial labyrinth [6,8,9,10]. This structure has been described in detail using computed tomography imaging and histological preparations (Figure 1). I in dogs, which are macrosmatic animals, the turbinate system fulfills a dual functional role. While the caudal portions of the ethmoturbinates are covered by olfactory epithelium and contribute directly to odor detection, a substantial proportion of the dorsal, middle, and ventral nasal turbinates (nasoturbinates and maxilloturbinates) is lined with respiratory epithelium. These structures play a critical role in conditioning the inspired air through heating, humidification, filtration, and regulation of airflow dynamics. Such conditioning is essential for maintaining optimal physicochemical conditions for odorant solubilization and receptor activation in the olfactory mucosa, thereby indirectly supporting olfactory performance [6,9].

The surface area of the olfactory mucosa in the canine nasal cavity is extensive, and the bony lamellae of the ethmoid bone divide the inhaled air into separate streams. The nasal turbinates regulate airflow within the nasal cavity. During sniffing, a distinct fraction of inspired air is selectively directed toward the dorsal nasal meatus and ethmoidal region, enhancing odorant delivery to the olfactory epithelium [9,10].

### Aerodynamic Pathway of Inspired Air During Sniffing in Dogs

During sniffing, dogs generate a highly specialized nasal airflow pattern that differs fundamentally from that observed during quiet respiration. Inspired air enters through the external nares, whose mobile structure and associated musculature allow precise modulation of airflow direction and velocity. Morphometric studies combined with computational fluid dynamics (CFD) modeling have demonstrated that, already in the rostral part of the nasal cavity, the incoming airflow is divided into two functionally distinct streams: a respiratory stream and an olfactory stream [9,10].

The majority of inspired air (over 80–85%) is directed through the ventral and middle nasal meatuses toward the nasopharynx and subsequently to the lungs. In parallel, a smaller but functionally critical fraction of air (approximately 10–15%) is selectively diverted toward the dorsal nasal meatus and the ethmoidal region lined with olfactory epithelium. This functional segregation enables simultaneous respiration and odor analysis without mutual interference between these processes [9].

During active sniffing, airflow within the olfactory region is characterized by reduced velocity and increased turbulence, which enhances the residence time of odorant molecules in close proximity to the cilia of olfactory sensory neurons. Moreover, the complex three-dimensional architecture of the ethmoidal endoturbinates and ectoturbinates induces local vortices and flow separation, promoting efficient deposition of odorant particles onto the olfactory mucosa and thereby increasing olfactory sensitivity [6,9].

A distinctive feature of canine olfaction is the functional separation of inspiratory and expiratory airflow pathways. During expiration, air is expelled predominantly through lateral slits of the nostrils rather than retracing the inspiratory route. This mechanism prevents the displacement of odorant molecules from the olfactory epithelium during exhalation and allows continuous sampling of new odor cues with each successive sniff [9]. Such an arrangement significantly enhances the dog’s ability to detect, discriminate, and localize odor sources, particularly during tracking and search behaviors.

Sniffing with the mouth closed relies almost exclusively on nasal breathing and favors maximal separation between respiratory and olfactory airflow streams, resulting in optimal odorant delivery to the olfactory epithelium. In contrast, sniffing with the mouth open—commonly observed during intense physical activity or thermal stress—introduces an oronasal breathing pattern in which part of the ventilatory demand is met through the oral cavity. This reduces the volume of air directed toward the olfactory region and may transiently decrease olfactory sampling efficiency. Nevertheless, the structural specialization of the canine nasal cavity allows maintenance of olfactory function even when oral airflow partially supplements nasal respiration [6,10].

The olfactory system is capable of recognizing a greater number of odors than the number of receptor types it possesses, because olfactory receptors exhibit broad cross-reactivity and participate in a combinatorial coding strategy, in which each odorant activates a unique combination of receptor types, generating distinct and highly discriminable patterns of neural activity. This mechanism underlies the enormous discriminatory capacity of the olfactory system, allowing the recognition of far more odorants than the number of receptor types alone would suggest [11,12]. Each nostril contains separate chambers, and air and odorant molecules do not mix within the nasal cavity. This anatomical separation allows more precise analysis of inhaled air by each nostril independently and enables identification of the direction from which an odor originates. A specialized bony structure facilitates the retention of odorant particles in a localized area, thereby prolonging the interaction time between odorant molecules and receptors.

The nasal turbinates are lined with a richly vascularized mucous membrane that regulates temperature, humidity, and blood flow, thereby facilitating the transport of odorant molecules. In dogs, olfactory perception is divided between the right and left nostrils, representing another example of sensory lateralization alongside vision and hearing. The olfactory epithelium in dogs primarily covers the dorsal and caudal regions of the nasal cavity, including the upper surfaces of the turbinates and the nasal septum. It is composed mainly of neuroepithelium containing three principal cell types: olfactory neurons bearing hundreds of cilia on a single cell, which markedly increases sensitivity; supporting cells that provide metabolic and structural support; and basal cells that enable continuous epithelial regeneration throughout life [13,14].

Estimates suggest that the canine nasal cavity contains on the order of hundreds of millions of olfactory receptor neurons, commonly cited at approximately 220 million, based on classical anatomical and histological assessments [15,16]. The epithelium also contains Bowman’s glands, which produce mucus that enables dissolution and transport of odorant molecules to the receptors. This epithelium extensively lines the ethmoturbinates, the dorsal nasal septum, and the dorsal and middle turbinates, with its most caudal portions lying adjacent to the cribriform plate. Axons of olfactory receptor neurons traverse the foramina of the cribriform plate to reach the olfactory bulb [17]. The canine olfactory bulb represents the first center of olfactory processing in the brain. Located within the ethmoidal fossa, with the cribriform plate forming its rostral bony boundary, the olfactory bulb is characterized by the presence of glomeruli, which are structural units where axons of olfactory neurons synapse with mitral and periglomerular cells. The olfactory bulb exhibits a seven-layered structure (Table 1).

The olfactory bulb in dogs is characterized by a higher volume-to-density ratio of glomeruli compared with humans and other mammals, reflecting an expanded receptor space. The olfactory bulb operates via mitral cells that transmit encoded signals to higher brain structures, such as the olfactory cortex and telencephalic nuclei, enabling precise odor discrimination and localization [19].

Interestingly, in older dogs a reduction in the number of olfactory receptor neurons (ORNs) within the olfactory epithelium has been observed in histopathological studies. In addition, the number of cilia on neurons responsible for capturing odorant molecules is reduced. Degeneration of the mitral cell layer and the periglomerular cell layer within the olfactory bulb has also been reported [20].

Resting-state functional magnetic resonance imaging (fMRI) studies have demonstrated that in older dogs (over 8 years of age) the strength of functional signals between the olfactory bulb, the hippocampus, and the frontal cortex is decreased. In particular, reduced integration of the olfactory–limbic network has been observed, which is responsible for associating odors with emotions and memory. Functional connectivity with the cingulate gyrus, a region involved in reward valuation, was also diminished [21].

## 3. Neurobiology of Odor Perception

Olfactory perception begins with the activation of olfactory receptors located in the olfactory epithelium (olfactory mucosa). Each receptor responds to a specific group of odorant molecules, and this information is encoded by the pattern of activated receptors [13,22]. Olfactory receptors represent a class of exteroceptive chemoreceptors that respond to chemical stimuli present in the air. Receptor proteins located in the membrane of the cilia bind specific odorant molecules, thereby initiating the process of signal transduction.

When an odorant molecule reaches a cilium, it binds to an olfactory receptor protein (OR), triggering a G-protein–mediated signaling cascade. Activation of the G protein (Gα_olf_) stimulates olfactory-specific type III adenylyl cyclase (ACIII), which catalyzes the conversion of adenosine triphosphate (ATP) to cyclic adenosine monophosphate (cAMP) [23,24]. An increase in cAMP concentration opens cyclic nucleotide–gated (CNG) ion channels, allowing sodium ion (Na^+^) and calcium ions (Ca^2+^) to enter the cell. This ionic influx causes depolarization and generates a receptor potential. The entry of Ca^2+^ ions further activates chloride channels, resulting in the efflux of chloride ions (Cl^−^) from the cell, which amplifies depolarization; the chloride current thus acts as a signal amplification mechanism. If the receptor potential reaches threshold, the olfactory receptor neuron generates an action potential that propagates along its axon [25,26].

Stronger stimuli induce a higher frequency of action potentials proportional to stimulus intensity. Suprathreshold stimuli result in increased firing frequency, whereas maximal stimulation does not further increase firing rate, leading to saturation of the action potential response. The impulse generated by the receptor neuron is transmitted to the olfactory bulb, where axons of ORNs form excitatory synapses primarily with the apical dendrites of second-order projection neurons—mitral and tufted cells—within structures known as glomeruli. At this stage, preliminary analysis and filtering of olfactory information occur, organized according to receptor type. Each receptor type detects specific groups of chemical compounds, and ORNs expressing the same receptor converge onto the same glomeruli in the olfactory bulb [27,28,29,30].

Glomeruli are not randomly distributed but are spatially organized according to receptor type, forming a so-called olfactory (glomerular) map—a topographic representation in which odor identity is reflected by distributed patterns of glomerular activity. Each odorant activates a distinct combination of receptors and, consequently, a specific pattern of glomerular activity. The brain interprets these spatial patterns as particular odors, analogous to reading a chemical barcode. Inhibitory and excitatory mechanisms within the olfactory bulb enable the selection of relevant signals while suppressing background noise. Periglomerular and granule cells act as local interneurons that modulate the activity of mitral neurons transmitting information to higher brain centers. This organization allows for selective transmission, amplification of biologically significant odors, and attenuation of interfering stimuli [13,31,32,33,34,35].

### Neuromodulatory and Hormonal Influences on Olfactory Processing in Detection Dogs

Olfactory processing in dogs is not limited to peripheral receptor activation and early sensory coding in the olfactory bulb but is dynamically modulated by neuromodulatory and hormonal systems that integrate sensory input with motivational state, reward expectation, learning, and memory. Among these systems, dopaminergic signaling plays a central role in shaping olfactory-guided behavior, particularly in working and detection dogs.

Dopaminergic projections originating from mesencephalic structures and forebrain nuclei modulate activity within the olfactory bulb, piriform cortex, limbic structures, and frontal cortical areas. Dopamine influences synaptic plasticity at multiple levels of the olfactory pathway, regulating the gain of sensory signals, enhancing signal-to-noise ratio, and selectively reinforcing odor representations associated with positive outcomes. This neuromodulatory control allows biologically or behaviorally relevant odors to be preferentially processed and retained in memory, while irrelevant stimuli are attenuated.

In detection dogs, olfactory cues are tightly linked to reward-based learning paradigms, in which correct identification of a target odor is consistently paired with reinforcement. Dopaminergic signaling is critically involved in reward prediction and reinforcement learning, strengthening neural representations of target odors through experience-dependent plasticity. Repeated pairing of a specific odor with reward leads to long-term changes in olfactory bulb circuits and higher-order olfactory and limbic regions, facilitating rapid recognition, improved discrimination, and sustained motivation during search tasks.

Neuromodulators also interact with hippocampal and limbic networks responsible for associative learning and memory consolidation. Functional connectivity between the olfactory bulb, hippocampus, amygdala, and prefrontal cortex enables the integration of olfactory information with contextual, emotional, and motivational cues. Hormonal influences, including stress-related glucocorticoids, can further modulate olfactory sensitivity and learning efficiency, either enhancing odor memory formation under moderate arousal or impairing performance under excessive stress.

Together, these neuromodulatory and neuroendocrine mechanisms transform olfactory perception from a purely sensory process into a flexible, experience-dependent system optimized for goal-directed behavior. In trained detection dogs, the close coupling between olfactory processing, dopaminergic reward circuits, and memory systems underlies their exceptional performance, persistence, and reliability in complex detection environments [3,4,19].

## 4. Integration of Odor with Memory and Emotions

Studies using diffusion tensor imaging magnetic resonance imaging (DTI MRI) and anatomical dissection with the Klingler method have demonstrated that at least five major white matter bundles (tracts) originate from the olfactory bulb (Table 2). These tracts connect the olfactory bulb with various brain structures, including the limbic system (encompassing the amygdala and hippocampus), the frontal lobes, and the visual cortex, via the olfactory tract projecting to the occipital lobe [36].

Mechanisms of learning within the odor–emotion–response sequence operate dynamically through the amygdala, which assigns emotional value, and the hippocampus, which encodes episodic memory. Behavioral studies have demonstrated that odors can evoke memories in dogs. Dogs remember tasks conditioned by the presence of a specific odor and perform better when that odor is reintroduced [37,38].

Dogs are capable of recognizing human emotions through odor, including stress, fear, and joy. Experimental studies show that dogs respond more strongly to human stress-related odors originating from breath, mucus, and sweat, which in turn influences their behavior and learning processes. It has been demonstrated that exposure to human stress odor reduces dogs’ propensity for exploration in cognitive tasks [39,40]. During intense emotional experiences, humans emit characteristic odors that are detected by dogs, thereby affecting canine behavior and cognitive processing.

Together with evidence that odors act as powerful cues for memory retrieval, these findings illustrate a strong functional integration between olfaction, memory, and emotions in dogs [1]. Odors can modulate stress and anxiety levels or encourage exploratory behavior, which is of particular importance in training, therapeutic contexts, and human–dog interactions. Dogs identify their owners and differentiate emotional states of humans based on chemical signals. Olfactory activity is a natural and species-typical behavior in dogs and represents a crucial component of animal welfare. Limiting opportunities for sniffing may lead to frustration, stress, and even behavioral problems [41,42].

## 5. Molecular and Genetic Aspects of Olfaction

Early canine genome analyses identified 971 olfactory receptor (OR) genes and suggested that this represented ~80% of the repertoire, with a relatively low pseudogene fraction (approximately ~12–18%) [7,43]. To avoid overlapping ranges and improve clarity, we follow a consolidated estimate supported by broader comparative mammalian annotations: dogs are currently estimated to possess approximately ~1100–1300 total OR genes, with ~15–20% pseudogenes, yielding roughly ~800–1000 functional OR-coding genes [44,45]. In comparison, humans have a much higher pseudogene proportion (commonly reported at >50%), resulting in markedly fewer functional OR genes; consequently, the functional OR repertoire in dogs remains approximately ~2–3 times larger than in humans [7,44].

The evolution of the OR gene family follows the so-called “birth-and-death” model, in which new genes arise through duplication, whereas others undergo pseudogenization and loss of function [45]. This process is influenced by both environmental pressures and factors related to species use, including training and behaviorally directed selection [46]. The greater number of olfactory receptor genes in dogs translates into an expanded potential for odor detection, allowing perception of a broader spectrum of odorant compositions. Although the number of coding genes is not the sole determinant of olfactory performance—neuronal architecture also plays a significant role—the receptor repertoire provides the molecular foundation for the higher sensitivity and discrimination abilities observed in dogs [7,43,47,48,49].

Comparative analyses among dogs (*Canis familiaris*), wolves (*Canis lupus*), and coyotes (*Canis latrans*) suggest that differences in olfactory capabilities are driven more by behavior and ecological strategies than by the absolute number of receptor genes alone [47,50,51]. These findings indicate that both molecular factors, such as the number and functionality of olfactory receptor genes, and environmental and behavioral influences jointly shape olfactory perception in dogs.

## 6. Evolution of the Sense of Smell

In vertebrates, olfactory sensory neurons (OSNs) originate from the olfactory placode, a thickening of the ectoderm in the anterior region of the developing head. Peripheral tissues of the olfactory organ are also formed in part by neural crest cells, for example, those giving rise to olfactory glia. Peripheral tissues of the olfactory organ are also formed in part by neural crest cells, for example, those giving rise to olfactory glia. This developmental model, involving both the olfactory placode and the neural crest, is well established in developmental and comparative studies of vertebrate embryology. The placode gives rise to the olfactory epithelium as well as to neurons whose axons project to the olfactory bulb of the brain, where they form glomeruli, the fundamental units of odor mapping. Already at the embryonic stage, the VNO differentiates in some vertebrate lineages, initiating the division into the main and accessory olfactory systems [52,53,54].

A major evolutionary breakthrough was the emergence of a large family of genes encoding olfactory receptors—seven-transmembrane G protein–coupled receptors (GPCRs)—and their dynamic expansion across different lineages. Comparative analyses among 23 chordate species have shown that the transition from aquatic to terrestrial environments profoundly altered the spectrum of chemical stimuli and shaped the evolutionary trajectory of OR. Genomic studies indicate that in mammals the OR repertoire typically reaches around ~1000 genes. In many teleost (ray-finned) fishes, functional OR gene numbers are often lower (commonly ~100–200), but important exceptions occur among non-teleost ray-finned fishes (e.g., Polypteriformes), which can exhibit substantially expanded OR repertoires linked to complex multilamellar olfactory organs. In fishes, receptors are predominantly adapted to detect water-soluble molecules, whereas in tetrapods there was an explosive expansion of OR copies specialized for the detection of volatile odorants. This pattern is well described by the “birth-and-death” model: local duplications within OR gene clusters, often occurring in tandem, generate new copies that are either retained under directional selection by acquiring novel sensitivities or rapidly undergo pseudogenization. The rate of these cycles is strongly environment-dependent. Macrosmatic rodents and carnivores, including dogs, which explore complex environmental cues such as tracks, prey traces, and social signals, exhibit rich and dynamic OR subfamilies. In contrast, lineages that have secondarily adapted to aquatic life, such as cetaceans, as well as primates in which vision plays a relatively greater role, accumulate pseudogenes and show reductions in OR subfamilies responsive to volatile odors [55,56,57].

In parallel, the accessory (vomeronasal) system diversified. In dogs, the VNO is retained and shows morphological and immunohistochemical features consistent with a functional accessory olfactory system [58]. However, genomic data indicate a partial receptor-level regression: the vomeronasal type-1 receptor (V1R) repertoire is markedly reduced (approximately ~8 intact genes, with many pseudogenes), and the vomeronasal type-2 receptor (V2R) family is fully degenerated (no intact copies; only pseudogenes reported), consistent with lineage-specific turnover in vomeronasal receptor families [59,60,61]. In reptiles and most small mammals, there was an expansion of the V1R and V2R receptor families associated with social and reproductive communication, whereas in some primates and certain aquatic mammals, particularly marine species, this system underwent regression. In amphibians, which occupy both aquatic and terrestrial environments, the receptor repertoire remains mosaic, combining features adapted to both habitats [62,63].

Genomic changes were coupled with reorganization of the olfactory brain. An increased number of functional ORs promoted the formation of more numerous glomeruli in the olfactory bulb and a more fine-grained mapping of odor quality. In early synapsids (mammal-like reptiles) and mammals, complex nasal turbinates evolved, increasing epithelial surface area and odorant contact; this adaptation is also associated with endothermy and nocturnal activity. Additional microevolutionary innovations, such as the ancient but relatively small family of trace amine–associated receptors (TAARs), which detect monoamines and biogenic amines signaling danger or food-related information, further complemented the system. Together, these features produced a multilayered, environmentally responsive olfactory system that in mammals achieved exceptional chemical resolution [33,64,65,66].

## 7. Evolution and Development of the Olfactory Organ in Canids

The evolution of the olfactory organ represents a clear example of adaptation to terrestrial life, in which the perception of airborne odors conferred a strong selective advantage. Although data specific to Canidae remain limited, available evidence suggests that genetic adaptations have resulted in an exceptionally diverse receptor repertoire in species that rely heavily on olfaction [67].

Recent integrative studies combining anatomical and genetic approaches across 56 domestic dog breeds indicate a reduction in the anatomical complexity of the olfactory system as a consequence of domestication when compared with wild ancestors [68]. Numerous studies have demonstrated substantial adult neurogenesis within the olfactory bulb, a phenomenon well established in rodents and other model species. In dogs, direct in vivo evidence remains limited; however, multipotent neural progenitor cells have been successfully isolated from the adult canine olfactory bulb and shown to differentiate into neurons, astrocytes, and oligodendrocytes in vitro, supporting the presence of latent neurogenic potential [69]. Together with comparative mammalian data, these findings suggest that olfactory bulb neurogenesis contributes to neural plasticity and olfactory learning capacity, albeit with species-specific differences in magnitude [70,71,72].

The evolution of canids has produced highly refined adaptations of the olfactory system, evident in the expansion of olfactory receptors distributed across elaborate nasal turbinates and in rich neuronal connectivity. Domestication-related changes also extend to the accessory olfactory system, as comparative neuroanatomical studies indicate partial reduction in vomeronasal structures in domestic dogs relative to wild canids [68]. Domestication has influenced a partial reduction in the accessory olfactory system VNO in dogs. Although the canine VNO is morphologically and immunohistochemically consistent with a functional organ [58], genomic analyses demonstrate a markedly reduced vomeronasal receptor repertoire, including only ~8–9 intact V1R genes and a complete degeneration of the V2R family, with exclusively pseudogenized copies [60,61]. In contrast, the MOS has been preserved and remains extensively exploited in practical and applied contexts. Future research integrating genomic data on OR genes, comparative anatomy, microbiome composition, and environmental factors will be essential for a more comprehensive understanding of the evolution and functional optimization of the olfactory organ in canids.

## 8. The Role of Natural and Artificial Selection

The olfactory system of the domestic dog is the product of a complex evolutionary process shaped by both natural and artificial selection pressures [73]. During domestication and selective breeding, humans deliberately influenced anatomical, neurobiological, and behavioral traits in dogs, thereby also affecting the structure and performance of the olfactory system. Natural selection acting on the ancestors of domestic dogs favored the development of efficient olfactory capabilities essential for food acquisition, spatial orientation, and social communication.

Artificial selection, intensively applied by humans over thousands of years, has exerted a more heterogeneous influence on olfactory abilities. In working breeds, selection has preferentially favored individuals combining high olfactory aptitude with behavioral traits that support sustained, goal-directed search performance and efficient reinforcement learning. In contrast, in companion, ornamental, and brachycephalic breeds, breeding preferences were largely focused on external appearance.

### Behavioral Phenotypes Shaped by Artificial Selection in Working Dogs

Artificial selection has shaped not only canine cranial and olfactory anatomy but also behavioral phenotypes that determine whether olfactory potential translates into reliable operational performance. In working contexts (e.g., detection, police, search, and competitive obedience), breeders and training organizations typically prefer dogs characterized by high search motivation (often conceptualized as play or prey drive), persistence, resilience to distraction, low fearfulness, and rapid reinforcement learning. These traits support sustained, repetitive sampling behavior and continued task engagement under variable environmental conditions, which is critical for operational reliability [4,41].

Selective breeding for working roles is therefore rationalized as a form of applied behavioral optimization: it aims to increase the likelihood that individuals will display stable, predictable responses to training cues, high reward responsiveness, and consistent task execution across handlers and contexts. In detection and police work, operational outcomes depend on a dog’s ability to maintain a focused search pattern, tolerate novelty and stressors, and show clear, repeatable indication behaviors. Empirical evidence indicates that performance in drug detection varies by breed, training level, target substance, and search environment, emphasizing that selection and training interact to produce functional outcomes rather than olfactory anatomy alone [3].

Importantly, selection criteria can influence behavioral consistency at the population level. Breeding programs that systematically prioritize working temperament—such as engagement with the handler, willingness to initiate and sustain search, and balanced arousal—tend to yield more homogeneous behavioral profiles, facilitating standardized training pipelines and reducing variability in field performance. Conversely, when selection focuses primarily on morphology or companion traits, the resulting behavioral heterogeneity can increase the probability of mismatch between a dog’s motivational profile and operational demands, even when baseline olfactory capacity is high [4].

Neurocognitive factors relevant to working performance also align with selective pressures. Detection tasks are commonly structured as reward-based learning paradigms; thus, behavioral traits that support efficient memory consolidation and stable performance under arousal are operationally advantageous. Experimental work in detection dogs suggests that arousal levels during and after training can modulate memory consolidation, implying that selection for appropriate arousal regulation may contribute to long-term training success and behavioral reliability [40]. Together, these observations support the concept that artificial selection for working roles operates on an integrated phenotype—combining olfactory anatomy, cognitive-emotional regulation, and trainability—to maximize behavioral consistency and operational performance. These findings emphasize that artificial selection in working dogs primarily targets behavioral and cognitive traits supporting reliable task execution, rather than direct enhancement of the olfactory receptor gene repertoire.

Contemporary fMRI, DTI and histological studies confirm that significant anatomical and functional differences in the olfactory system exist among breeds and arise directly from selective breeding decisions [2,67].

In the study by Mouton et al., 2025 [67], morphometric and genetic data from 56 dog breeds were analyzed, revealing a strong correlation between skull morphology and genetic markers associated with the olfactory system. The authors demonstrated that intensive selection toward a flattened facial skeleton, characterized by a shortened muzzle and reduced nasal cavity, correlates with anatomical constraints affecting the olfactory system. Importantly, no evidence was found for breed-specific selection favoring enhanced olfactory receptor gene expression or for differences in the Functional Olfactory Repertoire Gene (FORG) set between scent hounds and other breeds [67]. These results suggest that reduced olfactory performance observed in some brachycephalic breeds is more likely attributable to morphological and airflow-related constraints than to direct genetic depletion of the olfactory receptor repertoire. A reduced total surface area of the olfactory epithelium and impaired airflow delivery directly decrease odor detection efficiency. Furthermore, altered skull morphology in brachycephalic breeds is associated with changes in the spatial orientation and anatomical embedding of the olfactory bulbs, which may indirectly affect odor processing efficiency and integration, although direct evidence for altered functional connectivity with limbic or frontal regions in dogs remains limited. This translates into poorer integration of olfactory cues with memory and emotional processing [74,75,76,77].

Comparable conclusions were drawn by Bird et al., 2020 [50] regarding the impact of domestication on olfactory function. Using computed tomography and digital morphometric measurements, the authors quantified nasal turbinate surfaces in 46 domestic dog breeds (*Canis familiaris*), gray wolves (*Canis lupus*), and coyotes (*Canis latrans*). They showed that relative cribriform plate surface area (RelCP) correlates with components of the olfactory system but serves only as an indirect marker, as surface area alone does not directly predict olfactory performance. The authors suggested that domestication and subsequent breed specialization may have led to partial regression of olfactory function, which in some breeding lines may be irreversible without the introduction of alternative genetic pools [50].

Dolichocephalic dogs exhibit the highest olfactory network coherence and the shortest detection times in odor discrimination tests, further supporting the link between cranial morphology and olfactory performance [6,21].

## 9. Adaptation to Tracking, Hunting, and Cooperation with Humans

When discussing the origin and domestication of the domestic dog, it is essential to address a behavioral framework inherited from wolves: the so-called predatory motor pattern (predatory sequence). This concept provides a foundation for understanding hunting-related behaviors in dogs. The predatory sequence consists of a series of successive behaviors: environmental orientation → visual fixation → stalking → chase → grab-bite → kill → dissection → consumption. In domestic dogs, artificial selection has disrupted and restructured this sequence: some elements have been enhanced, others weakened or suppressed, in order to adapt behavior to specific forms of work alongside humans. Genetic and behavioral reviews emphasize that a substantial proportion of inter-breed variation concerns tasks rooted in elements of the predatory sequence, such as herding, pointing, tracking, and retrieving, and that these differences have a heritable component [78].

Recent attempts to formally describe a canine “ethogram” of this sequence indicate that, despite domestication, the general structure of the predatory pattern remains recognizable; however, individual modules vary in frequency and intensity of expression depending on functional type. It is this modulation—rather than the emergence of entirely new behaviors—that best explains the diversity of breed specializations [79]. Herding breeds provide a clear example. In Border Collies and related types, selection favored the segments “orientation → visual fixation → stalking → chase,” while simultaneously suppressing grab-bite and killing behaviors toward livestock. Behavioral and genetic data consistently show that herding represents a redirected use of the predatory sequence without its terminal, consumptive stages. Recent genomic analyses of herding dogs indicate selection acting specifically on specialization within these early components of the predatory sequence [80,81].

Pointing breeds (pointers, setters) emphasize prolonged visual fixation and the immobile “point,” effectively arresting the sequence prior to the chase, thereby maximizing human control over game. In contrast, hounds and terriers were selected for sustained pursuit, strong grab-bite, and killing behavior, increasing effectiveness in hunting small prey. Retrievers represent yet another variant, characterized by an enhanced grab without damage (the so-called “soft mouth”) and the carrying of game to the handler, with the killing phase suppressed. Reviews of inter-breed behavioral differences synthesize these patterns, demonstrating consistent trait profiles aligned with the historical working roles of specific breed groups [79,82].

A distinct category comprises livestock guardian dogs, in which most elements of the predatory sequence toward domestic animals have been suppressed. These dogs are expected to remain vigilant and territorial without initiating chase or bite. Studies of herding–guardian breeds (e.g., Can de Palleiro) reveal predictable differences in chase tendency, reactivity, and fearfulness, illustrating how selection balances predatory drive with other traits desirable for the dog’s working role [83].

From a human safety perspective, it is important to distinguish emotional aggression from predatory attacks. Clinical and review studies indicate that so-called “predatory attacks,” although rare, are weakly associated with the emotional arousal typical of other forms of aggression. Instead, they reflect activation of the predatory sequence, whereas emotional aggression has a strong communicative component and is accompanied by sympathetic nervous system arousal. This distinction is critical for both diagnosis and prevention strategies [84].

In relation to scent work performed for human purposes—such as explosive and narcotics detection, search and rescue, biological detection, and conservation tasks—the literature consistently emphasizes that detection success is primarily determined by behavioral traits. These include a strong motivation to search for odor, persistence in searching, high reward value (most commonly toys), independent task engagement, and resistance to distraction. Detection dogs must maintain activity over extended periods, often under challenging environmental conditions, without loss of motivation. Crucially, effective performance requires the “disconnection” of terminal stages of the predatory sequence (killing) while strongly reinforcing early modules such as environmental orientation, odor tracking, and “olfactory pursuit” toward the odor source [85,86].

Training practice capitalizes on these properties by establishing a contingency in which odor localization is immediately followed by access to a high-value reward (play, retrieving). This effectively “switches” the predatory sequence from consumption to substitute behaviors such as retrieving or tugging. The reward functions as a surrogate “prey object” upon which the predatory sequence terminates. In this way, the natural motivational tension associated with prey seeking is resolved in a safe and controlled manner.

One of the key characteristics of an effective detection dog is independence during work. In public safety contexts, such as explosive detection, the dog cannot rely on handler cues but must instead demonstrate initiative and autonomously explore the environment. Research shows that dogs with a natural propensity for independent odor tracking achieve superior outcomes in selection tests [85]. Breeds historically selected for hunting or retrieving (e.g., retrievers, spaniels) often yield individuals with desirable profiles, combining strong predatory drive and persistent search behavior with a low tendency toward destructive biting. Herding breeds (e.g., Border Collies) may excel in tasks requiring heightened orientation and fine motor control during searching, which can be advantageous in activities demanding precise, systematic scanning of odor plumes. At the same time, studies demonstrate substantial individual variation even within breeds, and standardized selection tools continue to be refined [86].

In dogs, the wolf-derived predatory sequence has not disappeared but has been reorganized. Human-directed selection stabilized and retuned those modules that enhance task utility—from the pointing behavior of gundogs, through the hyper-controlled motor patterns of herding dogs, to the sustained carrying behavior of retrievers—while suppressing undesirable elements such as killing. Recent genomic data from herding dog populations provide direct evidence that these very aspects of the predatory sequence were targets of selection, thereby closing the conceptual loop between ethology and the genetics of working performance [81].

## 10. Domestication and Breeding as Determinants of Olfactory Functions and Traits

Studies by Ortiz-Leal et al., 2022 [68], compared the neuronal structure of the olfactory organ in domestic dogs (*Canis familiaris*), red foxes (*Vulpes vulpes*), and gray wolves (*Canis lupus*). The authors demonstrated that all three species exhibit well-developed neurons in the glomerular and mitral layers of the olfactory bulb. However, foxes and wolves show a significantly larger relative olfactory bulb size in relation to other brain structures. Moreover, wild canids display a higher neuronal density in the periglomerular and mitral layers, as well as increased expression of neurochemical markers. These findings support the hypothesis that domestication has altered the structural organization of the olfactory system in dogs; reductions in relative olfactory bulb size and cellular density may constrain olfactory processing efficiency compared with wild ancestors, although functional outcomes are likely context- and breed-dependent [68].

A similar reduction has been observed in Jacobson’s organ (VNO), which mediates pheromone reception. Comparative neuroanatomical analyses of domestic dogs, wolves, and foxes indicate a partial reduction in vomeronasal structures in domesticated canids relative to wild counterparts [68,87]. These changes are consistent with altered selective pressures during domestication, although their precise functional consequences for intraspecific chemical communication remain to be fully resolved. More detailed comparative analyses of purebred dogs, village dogs, and wolves show that the selection of breeding individuals has acted on OR genes in different ways across successive stages of domestication and breeding. The study by Chen et al., 2012 [88] demonstrated that segregating OR pseudogenes in purebred dogs are subject to strong purifying selection, whereas in freely breeding dogs many of these loci evolve in a manner closer to natural conditions. In wolves, by contrast, deleterious variants tend to be eliminated while neutral polymorphisms are maintained, suggesting a balance of selective pressures in wild populations that differs from that observed in domesticated animals [88].

At the same time, numerous review and population-based studies confirm that breed formation—characterized by small effective population sizes, strong genetic bottlenecks, and directional selection—has reduced nucleotide diversity relative to village dogs. This process may weaken signals of selection at certain loci, while simultaneously facilitating the rapid fixation of advantageous variants in working lines. Through the international Dog10K project [89], which analyzed approximately two thousand whole genomes from purebred dogs, free-ranging dogs, and wolves, the scale of genetic differences among these groups has been precisely quantified. Dog10K analyses enabled the construction of detailed deoxyribonucleic acid (DNA) variation maps and the identification of so-called selection signatures—traces of natural or artificial selection in genes associated, among others, with sensory functions (such as olfaction and audition), behavior, and cognitive abilities.

Selection for olfactory working abilities also leaves detectable signatures over relatively short timescales. In working breeds, shifts in allele frequencies of specific olfactory receptor–related loci have been reported; however, available data do not indicate systematic selection for an expanded functional olfactory receptor repertoire. Instead, these patterns likely reflect indirect effects of selection acting on linked traits within constrained breeding timescales [90]. Furthermore, analyses linking genetic variants with behavioral traits in detection dog populations indicate that temperament, motivation, and exploratory drive are partly genetically determined and jointly influence olfactory performance. This demonstrates that superior olfactory ability in dogs arises from the interaction of both genetic–anatomical “hardware” and behavioral “software” [91].

Taken together, these findings support the hypothesis that there is no single universal genetic profile for olfaction across all dog breeds at the species level. Instead, differences in the olfactory apparatus are mosaic in nature, ranging from lineages in which selective pressure has been partially relaxed (e.g., extremely brachycephalic breeds) to lineages intensively selected for scent work, in which clear signatures of purifying and directional selection in olfactory receptor genes are maintained [67,89].

## 11. Differences in Olfactory Predispositions Among Dog Breeds

In the study by [92], olfactory abilities were examined in four groups of canids. The first group consisted of wolves, the second of scent hound breeds (Basset Hound, Beagle, German Pointer, Wire-Haired Vizsla, Bracco Italiano, Grand Basset Griffon Vendéen, Transylvanian Hound), the third of breeds considered non-scent-oriented (Bichon Havanese, Chinese Crested Powder Puff, English Greyhound, Hungarian Greyhound, Whippet, Afghan Hound, Bichon Bolognese, Greyhound cross, Miniature Pinscher, Siberian Husky), and the fourth of brachycephalic breeds (Cavalier King Charles Spaniel, Boston Terrier, Boxer, American Bulldog/Boxer cross, Bullmastiff, English Bulldog, Pug).

To address these differences, the authors developed an olfactory performance test—the Natural Detection Task (NDT)—which did not require prior training and assessed animals’ natural olfactory abilities. Samples of raw meat were placed in ceramic containers with openings. The test consisted of five difficulty levels. The first level was the easiest, with containers containing the largest number of openings, while each subsequent level had progressively fewer openings through which the odor could escape. Each individual completed four trials at each level, and correct indications were rewarded by access to the meat sample. The highest level at which performance remained above chance was recorded. Additionally, a selected subgroup (five wolves and seven dogs) was retested to assess measurement repeatability.

At the four easier difficulty levels, all dogs from all groups performed significantly above chance, confirming the validity of the test. At level five, only scent-oriented dogs and wolves achieved performance exceeding chance level; non-scent-oriented and brachycephalic dogs performed at chance level. Wolves did not initially outperform dogs, as their results in the first test were comparable. However, upon retesting, wolves significantly improved their performance, particularly at higher difficulty levels, reaching up to 90% success. Dogs selectively bred for scent work indeed demonstrated higher olfactory sensitivity, confirming the persistence of breed-related selection effects. Brachycephalic dogs performed worst, indicating a negative impact of shortened nasal anatomy on olfactory potential. The non-scent-oriented group showed intermediate performance, approaching chance level at the highest difficulty. The improvement observed in wolves only after repeated exposure may reflect differences in motivation, familiarity with experimental routines, or cognitive flexibility rather than purely genetic factors.

The NDT was shown to be a reliable tool, enabling rapid assessment of olfactory efficiency without prolonged training, making it valuable for the selection of dogs for scent work. The lack of improvement after the first test in dogs suggests that a single assessment may already be sufficiently informative. Because not all dogs prefer raw meat, the authors suggested repeating the test using commercial food to minimize variability in odor preference and intensity. The test should be conducted under controlled indoor conditions to eliminate confounding factors such as background odors, temperature, humidity, and airflow [92].

In a subsequent study, ref. [93] evaluated natural olfactory abilities across different dog breeds using the same NDT protocol. The study included 551 dogs (524 family dogs and 27 specialist explosive detection dogs), of which 484 were ultimately included in the analysis after excluding unsuccessful trials. Dogs were required to spontaneously indicate the odor source. Performance was assessed not by search speed but by reaction time at the highest level successfully detected by the dog (Successful Level Latency, SLL). Notably, Border Collies—a herding breed not primarily selected for scent work—achieved particularly strong results. In comparison, Golden Retrievers, Vizslas (Hungarian and German), as well as Bassets and Bloodhounds, showed statistically poorer performance, with longer reaction times and fewer successful indications.

Overall, younger and more energetic dogs performed better. Environmental factors, such as indoor versus outdoor testing conditions and ambient noise, also had a significant impact. Considerable individual variation was observed within the same breed. Genetic selection had not always been focused exclusively on olfactory abilities, and many breeds experienced a relaxation of specialist selection, reducing the predictability of olfactory traits. These findings highlight the importance of individual assessment rather than reliance on breed classification alone. Temperament, training level, and testing conditions proved critical, even in dogs with favorable genetic predispositions. Individual evaluation, particularly at an early stage, is therefore recommended when considering dogs for scent work. The NDT may serve as a useful selection tool, but even dogs from highly scent-oriented breeds require appropriate training and conditions to reach their full potential. Future studies should integrate interactions among genetic background (breed), environment, temperament, and training. Genetic or neurobiological investigations may further elucidate the mechanisms underlying differences in olfactory ability and motivation [93].

Selection in breeds developed for hunting and tracking was directly oriented toward scent-based tasks, shaping both their anatomy and behavior [50].

## 12. Practical Use of Dogs’ Olfactory Potential in Uniformed Services

Studies by [94] demonstrate the wide range of applications of canine olfaction in the detection of diseases, infections, emotional states, and even Coronavirus Disease 2019 (COVID-19), owing to the high sensitivity of the canine olfactory system, combined with dogs’ trainability and behavioral adaptability. In contrast to the limited number of studies conducted on wolves and other wild canids, the majority of current knowledge is based on data derived from domestic dogs. Dogs possess extraordinary olfactory capabilities (“macrosmia”), which are exploited in police work, search and rescue operations, medical detection, mold detection, hunting, and assistance for individuals with disabilities.

At the same time, an incomplete understanding of the biological and behavioral foundations of these abilities constrains their effective use and may negatively affect animal welfare. Dogs are capable not only of detecting extremely low concentrations of substances but also of discriminating between odorants that are chemically very similar. A more holistic approach is therefore required, integrating behavioral and neurological measurements and accounting for dogs’ emotional responses. Such an approach would improve understanding of the canine olfactory world and may contribute to the refinement of training methods, increased effectiveness in detection work, and enhanced animal welfare [41].

## 13. Classification of Service Dogs According to Their Functional Roles

The anatomy and physiology of the canine olfactory system enable the detection of odors in the form of volatile organic compounds (VOCs) at very low concentrations; controlled threshold studies report detection limits reaching the parts-per-trillion range [95,96]. Dogs are currently trained to detect substances relevant to rescue operations, medical diagnostics, environmental monitoring, forensic investigations, and many other applications [85,97,98].

### 13.1. Medical Detection Dogs and Assistance Dogs

Within this subsection, it is important to clearly distinguish between the different specializations encompassed by assistance dogs, as each category fulfills distinct functions and requires different selection, training, and validation approaches. Biodetection dogs are trained to recognize samples associated with specific conditions (e.g., infectious diseases or cancers) under laboratory or semi-field conditions. Alert dogs live with an individual and signal ongoing physiological changes in that specific person, such as hypo- or hyperglycemia, impending epileptic seizures, or anxiety episodes. Response assistance dogs intervene during an event (e.g., a seizure), provide safety-related behaviors, and may summon help. Each of these roles demands distinct recruitment criteria, training protocols, and validation procedures, including blind trials, controls for recognition of individual human odor, and assessment of generalization. Experts recommend systematic reporting of sensitivity and specificity, clearly defined success criteria, and regular retraining to maintain competencies [99].

Biodetection and medical alert dogs use olfaction to recognize specific profiles of VOCs associated with diseases or physiological states. Controlled experiments have demonstrated that dogs can discriminate samples from individuals infected with COVID-19 from negative samples across different biological matrices, including saliva, sweat, and urine. Importantly, generalization from inactivated to non-inactivated samples and across different bodily fluids has been shown, suggesting that infection is associated with a shared, global VOC pattern [100]. Field studies and systematic reviews report high, though variable, effectiveness of dogs in detecting infectious diseases and cancers, with reported sensitivities and specificities often ranging from 80% to 95%. These studies also emphasize the need for standardization and continuous retraining, particularly as pathogen-related odor signatures may change due to mutation. Pilot implementations have included airport screening applications [101,102,103].

Comparable conclusions emerge from studies led by Prof. Tadeusz Jezierski on cancer detection based on exhaled breath odor. Experiments involving lung and breast cancer yielded high sensitivities (approximately 85–90%) with moderate specificity. VOC analyses indicate that dogs respond to characteristic volatile profiles that partially overlap with markers identified using gas chromatography–mass spectrometry (GC–MS). These findings highlight the potential of dogs as highly sensitive “biosensors,” although full clinical implementation requires further validation, strict sample quality control, and clearly defined diagnostic protocols [104,105,106]. In addition to infectious and neoplastic targets, recent controlled experiments indicate that dogs can also detect acute human stress-related changes in VOC profiles. In a double-blind, three-alternative forced-choice test, dogs accurately discriminated paired breath and sweat samples collected from individuals before and after a stress-inducing task, indicating that stress responses are accompanied by detectable changes in VOC profiles. This capability is particularly relevant for assistance dogs supporting individuals with anxiety disorders or post-traumatic stress disorder [107].

Dogs alerting to metabolic disorders, such as hypoglycemia in diabetes - Diabetes Alert Dogs (DADs), have been reported to signal decreases in blood glucose levels under everyday living conditions. However, reported sensitivity varies widely across dog–handler pairs and depends strongly on methodology, with some data derived from owner-reported logs. Larger cohorts and studies independent of owner reporting confirm that many dogs exhibit clinically useful alert behaviors, yet results remain heterogeneous and underscore the need for standardized training, validation, and reporting criteria [108,109,110].

The literature also describes dogs that either respond during epileptic seizures or alert prior to seizure onset; both are generally classified as assistance dogs. While many reports are promising, including observations of reduced seizure frequency in some cases, robust evidence demonstrating accuracy beyond chance remains limited. Data on false-positive and false-negative alert rates under controlled conditions are still scarce. More recent studies seek to address these gaps, highlighting tangible benefits for many patients while emphasizing the necessity of standardized selection procedures and evaluation guidelines [111,112].

Overall effectiveness of medical and assistance dogs depends on sample quality, the risk of “handler odor carryover,” individual variability among dogs [4], and operational context (e.g., airport screening versus laboratory testing). Given the variability of outcomes, medical and assistance dogs should be considered complementary rather than substitutive to established diagnostic methods. Nevertheless, in selected applications—such as rapid screening or continuous monitoring of individuals—they may provide significant added value, improving safety and quality of life [103].

### 13.2. Patrol and Tracking Dogs (Patrol–Tracking Dogs)

Patrol dogs (patrol or dual-purpose dogs) are typically deployed within police or military units. They are trained for preventive and intervention tasks, including patrol duties, pursuit, and suspect apprehension. Tracking dogs (tracking or mantrailing dogs) specialize in following a continuous human scent trail in both open and urban environments, including search-and-rescue (SAR) and criminal investigations. The foundation of tracking work lies in the detection of VOCs originating from human skin cells, sweat, and the human microbiome, as well as from mechanical disturbance of the substrate. These mechanisms are described in studies on human scent profiles and olfactometry applied in forensic science [113,114].

Reviews and field studies indicate that trained dogs are capable of reliably locating and following the trail of a specific individual. Although results vary, success rates can be high under both controlled and operational conditions. Woidtke et al., 2018 [115] summarize that dogs can identify and track an individual based on personal scent, and that in many legal systems the results of their work are admissible as supporting—though not standalone—evidence [114,115]. Environmental conditions exert a significant influence: changes in temperature and humidity alter search strategies and the distance maintained from the scent trail. Dogs tend to track closer to the scent source under cooler and more humid conditions, whereas extreme weather changes may temporarily reduce performance. At the same time, some field analyses have not identified simple correlations with basic weather parameters, highlighting the complexity of tracking tasks and substantial variability among dog–handler teams [116,117,118].

Tracking can be divided into ground tracking (following ground disturbance and deposited scent) and mantrailing (following an airborne scent plume). In practice, these modes often overlap. Operational tasks include initiating a track from a scent article, maintaining the trail across heterogeneous and complex terrain (asphalt, sand, vegetation, buildings), and identifying the target individual at the end of the track (scent lineup). Researchers emphasize the necessity of training and testing under double-blind conditions (where neither the dog nor the handler knows the correct trail), controlling for contamination of scent samples, repetition of trials, and rigorous documentation of outcomes—particularly when results may carry legal consequences [114,119].

Patrol dogs also serve a deterrent function through their visible presence. In addition, they locate and apprehend suspects, support searches of buildings and vehicles, operate in nighttime conditions, and assist tactical intervention units. Practitioner accounts and operational reports often state that a single police officer with a dog may effectively replace several officers during patrol interventions. Criminological analyses and operational reports document both the effectiveness and the risks associated with using dogs for suspect location and apprehension (bite-and-hold or bark-and-hold methods). Large-scale studies show that the use of dogs can reduce escape attempts and resistance but is associated with a high incidence of injuries to suspects. These findings underscore the need for clearly defined procedures governing the deployment of service dogs, including proportional use-of-force principles, appropriate intervention methods, and systematic training and oversight of service dog (K9) teams [120,121,122,123].

In the Polish legal framework, the use of a service dog constitutes a means of direct coercion under the Act of 24 May 2013 on direct coercive measures and firearms; detailed provisions are specified in Article 21 of the Act. Accordingly, the service dog is classified as a distinct coercive instrument subject to the principles of legality, proportionality, and subsidiarity [124]. Comparable evidentiary standards and judicial scrutiny have been discussed in other jurisdictions, including Germany and the United States, where admissibility depends on documented reliability and validated procedures. The results of tracking dogs and dogs used for human scent discrimination may be admitted as auxiliary evidence, provided that training processes, proficiency testing, and documentation meet required standards of reliability and validity. Judicial assessment takes into account, in particular, the handler’s qualifications, the team’s performance history, error control procedures, and consideration of alternative explanations of results [114,119].

Tracking performance decreases with time elapsed since scent deposition, pedestrian traffic, adverse weather conditions (precipitation and strong wind), and in environments characterized by strong scent dispersion (e.g., street intersections, wind corridors in urban canyons). Studies on search dogs (SAR and conservation detection) have shown that dense and tall vegetation hampers detection and that target size affects both detection probability and the effective width of the search area. These findings are transferable to the tactical planning of patrol operations and tracking routes [125,126].

Patrol and tracking dogs represent complementary categories of service dogs: the former focus on security and intervention (often with a detection component), while the latter specialize in locating individuals or their traces based on olfactory cues. The effectiveness of both groups depends on appropriate dog selection and motivation, the quality of training and proficiency testing (including blind trials and contamination control), environmental conditions, and clearly defined rules for deployment and documentation—especially when canine performance has legal implications [114,118].

### 13.3. Detection Dogs for the Identification of Specific Odors

Detection dogs are employed in counterterrorism prevention, the suppression of smuggling, and the fight against organized crime. They constitute one of the most diverse categories of service dogs used in public security, forensic investigations, border protection, environmental monitoring, and medical applications. Their tasks include the detection of narcotics, explosives, weapons, banknotes, electronic devices, as well as biological traces (e.g., blood, semen, human tissues) and substances of animal or plant origin [86,127]. The literature emphasizes that, in many cases, the effectiveness of detection dogs equals or even exceeds the sensitivity of instrumental analytical methods, particularly under operational and field conditions [128]. In recent years, the scope of detection dog applications has expanded substantially, including environmental protection and ecology. Beyond traditional security-related tasks, detection dogs are increasingly used in environmental protection and ecology. For example, they are trained to locate feces of rare or endangered species for population monitoring, as well as to detect protected fauna and flora fragments [129].

The effectiveness of detection dogs depends largely on training quality and the maintenance of high work motivation. Odor learning is typically established through differential conditioning, in which exposure to a target odor (S+) is consistently paired with reinforcement, while non-target or distracting odors (S−) are not reinforced. In practical terms, this discrimination phase aims to build a robust association between the target odor and reward, while simultaneously training inhibitory control against common confounds (environmental distractors, handler-related cues, and non-target odorants). Controlled training formats (e.g., discrete trials or structured line-ups) can improve learning clarity by separating sampling, decision, and indication responses, thereby reducing ambiguity about which behavioral component is being reinforced [98,130,131,132].

Generalization training follows acquisition and is required because operational targets rarely occur as a single, fixed chemical stimulus. Dogs must generalize from trained exemplars to novel variants of the target odor (e.g., different formulations, concentrations, substrates, packaging materials, or mixtures), while avoiding overgeneralization to non-targets. Experimental work demonstrates that generalization gradients depend on training design and odor set structure; exposure to multiple exemplars during training and careful management of distractors can promote appropriate category formation and reduce both missed detections and false alerts under field conditions [131,132,133]. Contemporary studies increasingly employ standardized odor delivery devices and differential conditioning paradigms to improve repeatability and control odor concentration and presentation timing, which facilitates consistent learning and comparability across studies and operational programs [134].

Standardized protocols for odorant selection, storage, handling, and presentation are critical for training validity because dogs can learn not only “the target odor” but also any stable, unintended cues that covary with reinforcement. Deviations from technical standards—such as cross-contamination between target and distractor materials, variable container permeability, inconsistent headspace availability, residue transfer via gloves or tools, uncontrolled aging or evaporation, and non-randomized trial order—can introduce systematic bias. Such artifacts may shift learning toward irrelevant stimulus features (e.g., packaging, carrier materials, or contaminant odors), thereby reducing true target control and increasing false-positive indications when similar artifacts appear in operational contexts [1,86,128,130]. Methodological analyses of detection research emphasize that rigorous blinding, randomization, consistent odor storage conditions, and standardized presentation hardware are necessary to preserve reliability and interpretability of canine indications; failures in these areas can inflate apparent performance during training while degrading operational accuracy. Recent efforts to improve comparability between programs include the development of standardized calibrants and protocol recommendations intended to reduce inter-site variability and improve reproducibility of detection outcomes [86,130,135].

The performance of detection dogs is influenced by environmental factors (temperature, humidity, wind speed), the concentration and volatility of the odorant, as well as the dog’s experience, physical condition, and the handler’s skills [117,136,137,138,139]. Studies indicate that the stability of indications and the frequency of false alerts depend both on individual canine predispositions and on strict adherence to testing protocols and regular training. Standardization of training procedures significantly improves result reproducibility and reduces error rates [140,141].

At the same time, there is growing awareness of the importance of ethical standards and welfare considerations for service dogs, including minimizing stress exposure, ensuring appropriate working time, and maintaining positive motivation [142,143]. In modern security frameworks, detection-support technologies such as electronic noses (e-noses) are increasingly implemented. These systems do not replace dogs but rather enable preliminary screening, leaving final odor confirmation to biological detection teams [144,145].

### 13.4. Search and Rescue Dogs (SAR Dogs)

Search and rescue—SAR dogs are trained to locate missing persons following natural disasters, building collapses, and in open terrain. Their effectiveness is based on the detection of a complex human odor “signature” composed, among other components, of exfoliated skin fragments (so-called skin rafts) and VOCs originating from sweat, skin lipids, and water. These compounds can serve as markers even in challenging environments such as urban rubble. Reviews and experimental studies demonstrate that human odor exhibits an individually distinctive profile (a so-called odor “barcode”) that can be collected non-invasively for analytical purposes and for use in canine detection work [146,147,148].

In the context of Urban Search and Rescue (USAR), specific volatile markers of human presence have been proposed, with predicted concentrations in air pockets within rubble, helping to explain why dogs are able to indicate victims beneath layers of building materials. Research on the odor of human remains and bones shows that characteristic VOCs are detectable by trained dogs, which is also exploited in human remains detection (HRD) applications [149,150]. SAR dogs also operate in snow and avalanche conditions. Empirical studies document their ability to locate human scent beneath snow cover, as well as the influence of environmental factors such as fatigue, altitude, and cold on the efficiency and behavior of search teams [151,152]. In addition, specialized SAR teams trained for water search rely on the transport of human scent within the water column and above the surface; methodological studies and operational reports confirm the feasibility of odor-based localization from boats [153]. Selection and training of SAR dogs emphasize a specific behavioral profile, including high sociability, appropriate temperament, trainability, courage, low fearfulness, high energy levels, endurance, and physical fitness. Studies comparing certified SAR dogs with candidates (e.g., Federal Emergency Management Agency—FEMA programs) have shown that traits such as trainability, fearlessness, and high activity levels distinguish dogs that are successful in SAR work. Reviews on the selection of working dogs further highlight the importance of early testing—such as assessments of search behavior and fear reactivity at approximately three months of age—for predicting training success [154,155,156]. Accordingly, adaptability to novel and diverse environments (ruins, snow, forests, urban areas) is considered a fundamental requirement. Methodological research explicitly recommends training under conditions of variable odor availability and the standardization of training procedures in order to reduce false alerts and improve the stability of field performance. Long-term studies of SAR dogs suggest that resilience and well-designed training programs contribute to improved performance over time [130,157].

### 13.5. Dogs in Environmental Protection and Biomonitoring

The use of dogs in environmental biomonitoring is developing rapidly, as it combines the non-invasive nature of sample collection with the exceptionally high sensitivity of the canine olfactory system. A classic example is the use of so-called scat detection dogs, which locate feces of elusive or cryptic species. This enables the collection of genetic and hormonal material for analyses of population size, diet, stress levels, and parasite presence without the need to capture or disturb animals. Field studies demonstrate that trained dog–handler teams can achieve high detection efficiency and survey large areas more rapidly than human observers, while the collected samples provide critical information on species distribution and often determine whether conservation programs should be maintained. Review and analytical studies emphasize that carefully designed training and validation procedures, including blind trials and contamination control, are essential for the reliability of data obtained through this approach [158,159]. This approach has also been successfully adapted to marine biomonitoring, including the detection of floating cetacean feces (e.g., killer whales) to enable hormonal and toxin analyses; dogs can detect odor plumes at considerable distances under favorable wind conditions and guide vessels toward dispersed scent trails, increasing sampling efficiency compared with visual observation alone [160,161,162].

Dogs used in environmental protection also support early detection of invasive species. On land and in aquatic ports, they are successfully deployed to search boats, trailers, and equipment for quagga mussels (*Dreissena rostriformis bugensis*) and zebra mussels (*Dreissena polymorpha*), even at microscopic life stages and at minimal quantities of biological material. Their performance significantly exceeds that of visual inspections alone. Odor-based detection reduces inspection time and increases detection probability, which is critical for preventing unwanted introductions and minimizing ecological and economic costs associated with biological invasions [163,164]. In parallel, comparative studies increasingly evaluate canine detection alongside environmental DNA (eDNA) approaches, indicating that dogs provide rapid, field-based localization whereas eDNA can confirm presence at very low densities; the methods are therefore complementary within a robust biodiversity monitoring toolkit [165]. These studies indicate that dogs can respond more rapidly in the field and directly indicate the presence of a species, whereas eDNA analyses can confirm presence even at very low population densities. The two approaches are therefore complementary, forming a robust toolkit for biodiversity monitoring [165].

Another area of application for dogs with highly developed olfactory abilities is archeology. Dogs trained to detect the odor of human remains can assist in locating historical burial sites. Field studies suggest that this method has considerable potential, particularly in areas with heterogeneous soil structures where traditional search techniques may be less effective. At the same time, researchers stress that canine detection requires careful study design, including appropriate selection of test locations, collaboration with local communities and archeologists, and caution in result interpretation. Environmental factors, the age of remains, and preservation conditions can all influence odor presence. Review studies note that comparative research remains limited, particularly with respect to very old archeological finds. Nevertheless, well-trained dogs and experienced handlers can substantially improve and accelerate search processes, providing a valuable complement to classical archaeological methods [166,167].

## 14. Dog Specialization and Their Olfactory Profiles

The selection of an appropriate dog breed for a specific detection task plays a critical role in achieving high operational effectiveness. In domestic dogs (*Canis familiaris*), suitability for olfactory work is determined by a combination of innate sensory capacities, behavioral traits, and physiological characteristics. Research indicates that breeds selectively developed for scent-related tasks achieve significantly better performance in detection tests than breeds whose breeding history was not oriented toward olfactory work. In the experiment conducted by Polgár, scent-oriented breeds markedly outperformed non-selected breeds as well as brachycephalic breeds [92].

At the same time, recent studies emphasize that breed membership alone, or assignment to a specific breed group, is not a sufficient predictor of olfactory suitability. In the study by Salamon, although certain breeds achieved better overall results, no unequivocal relationship was found between breed group and olfactory test performance. Instead, individual characteristics—such as trainability and motivation for scent work—were identified as key determinants of success [168]. This individual variability extends to odor preferences and responsiveness, which may differ substantially even among dogs of the same breed [4].

When selecting a dog for a particular detection task—such as explosive or narcotics detection, human tracking, or environmental monitoring—it is therefore necessary to consider both breed-related tendencies and task-specific requirements. For tasks involving intensive tracking and work in terrain with uneven topography, breeds traditionally classified as scent hounds (e.g., Bloodhounds) are often preferred due to their elongated muzzle, high number of olfactory receptors, and strong scent motivation. For example, Polgár’s study demonstrated a clear advantage of scent-selected breeds over companion or brachycephalic breeds [92].

However, for many contemporary detection tasks, behavioral attributes may be more important than breed morphology alone. Traits such as high reward motivation, sustained concentration, resistance to environmental distractions, and responsiveness to training are increasingly recognized as primary determinants of task performance. Lazarowski and colleagues emphasized that these behavioral factors exert a stronger influence on detection success than the morphological characteristics associated with breed identity [130].

## 15. The Microbiome and Living Environment in Relation to Detection Task

An increasing body of evidence indicates that a dog’s olfactory profile is shaped not only by anatomical and physiological traits but also by the microbiome (both gastrointestinal and upper respiratory) and by living and working conditions, including diet, stress, temperature, humidity, and housing practices. Studies emphasize that the bidirectional gut–brain–olfaction axis modulates metabolism and neurochemistry, which may translate into olfactory behaviors and detection performance. Conversely, physical exertion, diet, and stress can alter the microbiome, thereby indirectly affecting olfaction [17,169].

Research on the microbiome of working dogs, including detection dogs, shows that differences in gut and mucosal bacterial composition co-occur with phenotypic traits relevant to work performance, suggesting a potential association with detection outcomes. At the same time, authors note that studies directly linking microbiome composition to detection efficacy remain limited, and that further research—such as dietary interventions, probiotic supplementation, and controlled olfactory testing—is required [170]. The nasal and oral microbiota of detection dogs varies across individuals and training environments, likely reflecting environmental influences and husbandry practices (e.g., hygiene, nutrition, microclimate). Although compositional variability alone does not determine performance, it highlights an important and previously underestimated dimension of controlling training and working conditions [171].

Environmental structure also affects odor transport and dispersion, as well as the physiology of sniffing. High temperatures, dehydration, associated dysfunctions of nasal mucus, and increased panting frequency reduce olfactory sensitivity and search efficiency. These parameters should be considered in task planning and performance interpretation [168]. From a methodological standpoint, standardization of training materials, control of environmental distractions, and double-blind training protocols are recommended to dissociate true changes in olfactory performance from handler influence [86].

For detection objectives such as identifying markers of infectious diseases, inflammatory biomarkers, or decomposition odors, volatile organic compounds (VOCs) are critical. VOC profiles may depend on both the health status and microbiome of the odor source and on environmental conditions. Understanding VOC origins and benchmarking canine performance against chemical analyses (e.g., GC–MS) can improve training design and the selection of training materials [113,172].

The gut and nasopharyngeal microbiome, together with living environment, thus constitute important factors influencing detection performance. Integrating nutrition, nasal hygiene, and stress management with rigorous standardization of working and testing environments should become a routine component of training programs [159]. Dysbiosis and disturbances of microbiota composition may reduce olfactory perceptual efficiency [17]. Growing evidence links microbiome composition and stability to behavior, stress reactivity, and cognitive functions in dogs, all of which directly impact working performance.

In a cross-sectional study of 134 dogs across different working categories, associations were identified between microbiome profiles and phenotypes relevant to work performance. The authors highlighted the potential of microbiome profiling as a source of performance indicators and suggested a link between microbiota and olfactory efficiency in detection dogs [170]. Biologically, such associations may be mediated via the gut–brain axis, influencing neurobehavioral and sensory processes, including olfactory processing [17,173].

Longitudinal observations suggest that under controlled training conditions, the microbiome of young dogs tends toward stabilization and resilience to environmental perturbations [174]. Short-term microbiome stability is particularly important for biomarker applications; recent analyses show low day-to-day variability in fecal microbiota composition in healthy dogs, supporting its use as a practical indicator of physiological state without frequent sampling in applied cynology (e.g., prior to field tasks) [175]. Service-related stressors such as transport and sensory overload influence bacterial composition; shifts have been observed following acute stress episodes, positioning the microbiome as a potential indicator of work readiness and post-exertion recovery [176]. Physiological changes associated with work in challenging conditions, such as heat exposure, are also reflected in microbiological parameters [177].

Using computational and data-driven approaches, researchers have reported correlations between microbiome composition and behavioral patterns such as anxiety and aggression, which may affect suitability for specific tasks. Microbiome profiles can predict behavioral group membership; for example, certain bacterial taxa (e.g., *Blautia*) occur more frequently in calm or well-adapted dogs, suggesting potential utility for predicting suitability for roles such as service, therapeutic, or detection work [178]. In working dogs, associations have also been reported between microbiome composition, aggression, and neurotransmission [179].

In young detection dogs, links have been observed between the microbiome and olfactory functions, sensory performance development, and cognitive capacities, including working memory. Authors propose that dietary modulation—through prebiotics, probiotics, and dietary fiber—could influence health and potentially olfactory performance by shaping the microbiome. Collectively, these findings support the hypothesis that the microbiome may modulate not only health and immune function but also sensory (e.g., olfaction) and cognitive (e.g., memory, attention) domains in working dogs [173,180].

### Stress, Arousal, and Olfactory Performance in Detection Dogs

Stress represents one of the most critical modulators of detection dog performance and welfare, acting through neuroendocrine, neurobiological, and behavioral pathways. From a functional perspective, it is essential to distinguish between eustress, defined as moderate, adaptive arousal that facilitates performance, and distress, which reflects excessive or chronic stress leading to performance degradation and compromised welfare.

At the neurobiological level, stress responses are primarily mediated by activation of the hypothalamic–pituitary–adrenal (HPA) axis and the sympathetic nervous system. Acute activation results in transient increases in catecholamines and glucocorticoids, which can enhance sensory alertness, attention, and memory consolidation when maintained within an optimal range. Experimental and applied studies in detection dogs demonstrate that moderate arousal during training can improve odor learning and memory stabilization, particularly when reward contingencies are clear and predictable [40,41].

This relationship follows an inverted-U function, analogous to the Yerkes–Dodson law, whereby eustress supports efficient olfactory sampling, sustained motivation, and rapid decision-making, while excessive arousal impairs these processes. Under conditions of acute distress—such as high thermal load, dehydration, unfamiliar noise, handler tension, or chaotic environments—dogs show reduced olfactory sensitivity, increased panting frequency that disrupts sniffing dynamics, and greater variability in indication behavior [137,139]. Behavioral manifestations include shortened search persistence, premature task disengagement, and elevated false alert rates.

Chronic stress exerts more profound and persistent effects. Prolonged activation of the HPA axis is associated with alterations in hippocampal and prefrontal cortical function, regions essential for memory, inhibitory control, and decision-making. In working dogs, chronic stressors such as excessive workload, inadequate recovery, transport, social instability, or suboptimal housing conditions have been linked to reduced learning efficiency, impaired discrimination performance, and increased behavioral reactivity [142,155]. Importantly, these effects may occur even when basic olfactory anatomy and receptor capacity remain intact.

Emerging evidence further suggests that stress-related changes interact with the gut–brain axis and the microbiome. Acute and chronic stress can modify gastrointestinal and nasal microbial composition, which in turn may influence neurochemical signaling, immune regulation, and cognitive performance. Studies in working and detection dogs report associations between microbiome profiles, stress reactivity, anxiety-related behaviors, and task performance, indicating that stress-mediated microbiome shifts may indirectly modulate olfactory readiness and learning capacity [170,176,178].

From an applied perspective, these findings underscore that optimal detection performance depends not on the elimination of stress, but on its regulation. Training paradigms that maintain dogs within a zone of functional arousal—characterized by high motivation, emotional stability, and predictable reinforcement—promote both performance reliability and welfare. Conversely, unmanaged distress compromises olfactory sensitivity, decision accuracy, and long-term usability of detection dogs.

Accordingly, contemporary best-practice frameworks emphasize the integration of welfare-oriented management, including appropriate workload scheduling, thermal mitigation, recovery periods, handler education, and monitoring of behavioral and physiological stress indicators. Such approaches not only protect animal welfare but also preserve the neurobiological conditions necessary for sustained high-level olfactory performance in operational environments.

## 16. Conclusions

The canine sense of smell is the outcome of a complex evolutionary history in which a high number of olfactory receptors, an extensively developed nasal turbinate structure, and strong neuronal connectivity have shaped one of the most effective biological detectors in the animal kingdom. The literature reviewed indicates that olfactory capabilities are not determined solely by the anatomical structure of the canine nose but result from the interaction of genetic, environmental, emotional, and behavioral factors consolidated through natural and artificial selection. Research suggests that domestication may have led to a partial loss of certain olfactory functions, particularly in brachycephalic breeds; however, this process has simultaneously given rise to lineages with exceptional detection specialization.

The widespread use of dogs in rescue, military, and civilian services confirms the high effectiveness of their work, provided that individuals are appropriately selected, trained, and maintained under conditions ensuring animal welfare. These findings underscore the need for further in-depth research aimed at assessing the influence of the nasal and gut microbiome on the quality of odor detection, as well as evaluating the potential of modern electronic devices capable of recording physiological parameters in dogs to improve methods supporting both the recruitment and training of service dogs.

## Figures and Tables

**Table 1:** Layers of the olfactory bulb. Author’s own elaboration based on classical histological studies of the canine olfactory bulb, including Miodoński (1968), and contemporary sources [17,18].

English Name	Latin Name	Description
olfactory nerve layer	stratum nervosum	contains axonal projections of olfactory sensory neurons originating from the nasal epithelium
glomerular layer	stratum glomerulosum	the site where axons of olfactory sensory neurons form synapses with the dendrites of mitral and tufted cells
external plexiform layer	stratum plexiforme externum	contains the somata and lateral dendrites of tufted cells, lateral processes of mitral cells, as well as amacrine and other interneurons
mitral cell layer	stratum cellulosum pyramidale	contains the cell bodies of mitral cells, which constitute the principal output neurons of the olfactory bulb
internal plexiform layer	stratum plexiforme internum	less distinct, containing fibers and synapses, including those of granule cells
granule cell layer	stratum granulosum	contains numerous interneurons involved in signal modulation
olfactory bulb core	centrum fibrosum	contains projection fibers and connecting elements linking the olfactory bulb with other regions of the brain

**Table 2:** Five tracts originating from the olfactory bulb, their connections, and functions. Author’s own elaboration based on studies by [36].

Abbreviation	Full Name	Main Connections	Function
OPT	Olfactory-Piriform Tract	piriform cortex	Odor Recognition and Olfactory Learning
OLT	Olfactory-Limbic Tract	amygdala, hippocampus	Emotional Responses and Emotional Memory
OET	Olfactory-Entorhinal Tract	entorhinal cortex	Episodic Memory and Spatial Orientation of Odors
OFT	Olfactory-Frontal Tract	frontal (prefrontal) cortex	Cognitive Decision-Making, Planning, and Odor Evaluation
OOT	Olfactory-Occipital Tract	occipital lobe (visual cortex)	Integrated Processing of Olfactory and Visual Stimuli

## Data Availability

No new data were created or analyzed in this study. Data sharing does not apply to this article.
